# Chronic Pain in Relation to Depressive Disorders and Alcohol Abuse

**DOI:** 10.3390/brainsci10110826

**Published:** 2020-11-07

**Authors:** Nasim Maleki, Marlene Oscar-Berman

**Affiliations:** 1 Department of Psychiatry, Massachusetts General Hospital, Harvard Medical School, Boston, MA 02129, USA; nmaleki@mgh.harvard.edu; 2 Psychology Research Service, VA Healthcare System, Jamaica Plain Campus, Boston, MA 02130, USA; 3 Departments of Anatomy & Neurobiology, Psychiatry, and Neurology, Boston University School of Medicine, Boston, MA 02118, USA

**Keywords:** chronic pain, headache, comorbidity, depressive disorders, alcohol abuse, alcohol dependence, alcohol use disorders

## Abstract

Chronic pain disorders have been associated separately with neuropsychiatric conditions such as depression and alcohol abuse. However, in individuals who suffer from non-cancer chronic pain disorders, it is not clear if the burden of depressive disorders is similar for those with and without a history of alcohol abuse. Using data from the Collaborative Psychiatric Epidemiology Surveys (CPES), we found depressive disorders to have a high burden in men and women with a history of alcohol abuse, independently of the presence or absence of chronic pain. We also found that, although the incidence of persistent depressive disorder was comparable in men and women with a history of alcohol abuse, and significantly higher than in control men and women, the incidence of a major depressive episode was higher in women with a history of alcohol abuse independently of the presence or absence of chronic pain. The age of onset of depressive disorders, independently of pain status, was younger for individuals with a history of alcohol abuse. The findings of this study have important implications for the clinical management of individuals who suffer from chronic pain comorbidly with depression and/or alcohol abuse.

## 1. Introduction

Chronic pain, depressive disorders, and alcohol abuse are widespread health conditions with a high risk for comorbidity [1,2,3,4,5]. In the U.S., an estimated 116 million adults suffer from chronic pain conditions [3,6]. Chronic pain may contribute to the risk of depression and alcohol abuse, and the associations can be bi-directional [4,7,8,9,10]. In the U.S., major depressive disorder affects about 13% of individuals in their lifetime. Prevalence estimates between 2016 and 2018 have shown that approximately 15 million Americans suffered from harmful use of alcohol [11], about 50 million from chronic pain disorders [12], and 17.3 million experienced at least one major depressive episode [13], and a lifetime prevalence of about 20% for major depressive disorder between 2012 to 2013, according to the National Epidemiologic Survey on Alcohol and Related Conditions (NESARC-III) [14].

Despite numerous reports on multi-directional associations between chronic pain disorders and depression or alcohol abuse [1,2,6,7,9,15,16,17], it is not clear if depressive disorders carry a different burden for those with and without a history of alcohol abuse in the presence of chronic pain. Consideration of the brain reward system may help to clarify the links among chronic pain, depression, and alcohol abuse by showing their overlapping neuroanatomy. For example, the dysregulation of brain reward circuitry may play a role in the interrelatedness of depression, chronic pain, and alcohol abuse [6].

**Figure 1:**
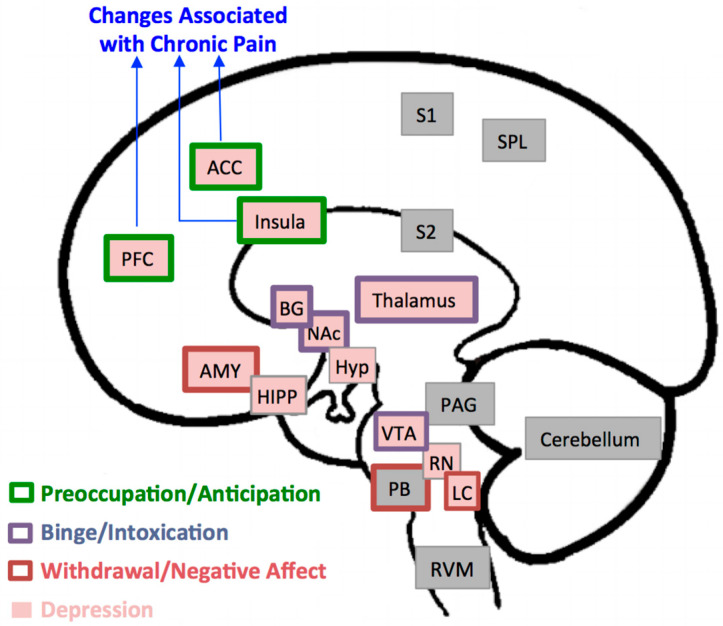
Brain Regions Implicated in Pain, Depression, and Alcohol Addiction. In this model, brain regions implicated in pain processing include the prefrontal cortex (PFC), anterior cingulate cortex (ACC), insula, thalamus, basal ganglia (BG), amygdala (AMY), parabrachial nucleus (PB), periaqueductal gray (PAG), rostroventral medulla (RVM), primary and secondary sensory areas (S1, S2), superior parietal lobe (SPL), and cerebellum [20]. Structural, functional, and metabolic changes in the first three regions, PFC, ACC, and insula, in particular have been consistently reported in association with chronic pain disorders. Brain regions associated with depression are the PFC, ACC, insula, thalamus, BG, AMY, hippocampus (HIPP), hypothalamus (Hyp), ventral tegmental area (VT), raphe nuclei (RN) and locus coeruleus (LC) [19]. The three stages of development and maintenance of addiction to alcohol and the regions associated with it [8] include: a binge intoxication stage (BG, thalamus, nucleus accumbens (NAc)); a withdrawal-negative-affect stage (AMY, PB, LC); and a stage of preoccupation-anticipation (PFC, ACC, insula).

Figure 1 illustrates brain regions involved in modulatory influences on descending pain pathways, nociception, and analgesia [18], in mood disorders and negative affect such as depression [19], and in the positive and negative reinforcing effects of alcohol [8]. Maladaptive neural changes in the brain’s reward system, especially in the prefrontal cortex, anterior cingulate cortex, and insula, are consistently reported in association with the exacerbation of chronic pain, alcohol abuse, and disorders of affect [6,8,9,19,20,21,22]. As such, abnormalities in those structures may provide a substrate for pain disorders, depressive disorders, and alcohol-related disorders to be manifested as comorbid conditions in vulnerable individuals.

Because of the interrelatedness among chronic pain disorders, depressive disorders, and alcohol abuse, and their common neural pathways, we hypothesized that in the presence of chronic pain, the burden of depression would be similar for individuals with and without a history of alcohol abuse. In other words, we expected that depressive disorders would be a high burden in patients with chronic pain, independently of whether or not they also have a diagnosis of alcohol abuse. To test this hypothesis, we leveraged a large national database [23], to compare the lifetime incidence of three depressive diagnoses in individuals with a history of alcohol abuse compared to those with no such history in the presence or absence of non-cancer chronic pain disorders. It should be noted that the data for the present study came from the Collaborative Psychiatric Epidemiology Surveys (CPES), which used DSM-IV diagnostic criteria of “Alcohol Abuse” and “Alcohol Dependence.” In DSM-V, those diagnoses were updated to integrate both Alcohol Abuse and Alcohol Dependence into a single diagnosis of “Alcohol Use Disorder.” Herein, we use the term alcohol abuse (ALC) to be consistent with CPES terminology for individuals meeting DSM-IV criteria for lifetime alcohol abuse, keeping in mind that approximately one third of the CPES cohort of ALC individuals also met DSM-IV criteria for lifetime alcohol dependence.

## 2. Materials and Methods

Sample: The CPES consists of three national studies conducted across the United States, with the support of the National Institute of Mental Health. The shared goal of these studies was to measure mental health symptoms and their severity, as well as to assess the utilization of healthcare services [24,25,26]. The CPES studies were conducted from 2001 to 2003, through in-person or telephone interviews with adults 18 years of age or older, and had an average response rate of 72.7% across the three studies [23].

Participants were asked to indicate whether or not they ever had chronic back/neck problems and/or frequent or severe headaches, and to specify if they had any other chronic pain anywhere else in their bodies. Individuals who answered “yes” to any pain condition also were asked about the age at which they first experienced each condition. Based on the answers to these questions, we selected three groups of people: (1) those with no history of any chronic pain, (2) those who had a history of only back and/or neck problems, and (3) those who had a history of severe or frequent headaches only. For Groups 2 and 3, in order to maximize homogeneity in each of the cohorts, individuals with a history of any other chronic pain disorder, multiple chronic pain disorders, medically unexplained pain, or arthritis/rheumatism were excluded.

Our analyses were based upon information provided by a total of 6765 individuals who had responded to questions related to chronic pain, depressive disorders, and alcohol abuse (ALC) and met the inclusion/exclusion of the study. Of those, 764 individuals (men: 528) met DSM-IV criteria for ALC. The remaining 6001 individuals (men: 2679) served as a control (CTRL) cohort, that is, individuals who had no current or past history of alcohol abuse or dependence, but who might have had a history of chronic pain and/or depression. The ALC cohort consisted of 528 individuals with no chronic pain history, 140 individuals with only chronic back or neck problems, and 96 individuals with only chronic or severe headache. The CTRL cohort consisted of 460 individuals with no chronic pain history, 690 individuals with only chronic back or neck problems, and 851 individuals with only chronic or severe headache.

Neuropsychiatric Conditions: Lifetime presence of three depressive disorder diagnoses were considered: major depressive episode (MDE), major depressive disorder with hierarchy (MDD), and dysthymia or persistent depressive disorder (PDD). The diagnoses were based according to DSM-IV criteria [27] and were available as part of the CPES dataset. In brief, MDE is characterized by depressive symptoms (depressed mood, loss of interest or loss of interest or pleasure) that have been present during the course of the same two-week period and represent a change from previous functioning. MDD is characterized by persistent or recurrent MDEs. Dysthymia or PDD is characterized by predominant depressed mood for more days and for most of any given day, for at least two years. We focused on these phenotypes because they are the most commonly reported depressive disorders in association with chronic pain disorders [28,29].

The presence of lifetime ALC was assessed as part of the CPES study [23] through structured interviews, using the framework of the World Health Organization’s Composite International Diagnostic Interview (CIDI) (3rd version [30]). The diagnosis of ALC in this assessment was based on the DSM-IV criteria [27]. According to these criteria, ALC is defined as a maladaptive pattern of alcohol use resulting in a failure to fulfill major personal, academic, or professional obligations; or resulting physically hazardous recurrent drinking; or resulting recurrent alcohol-related legal problems; or associated with misuse despite having social or interpersonal consequences. In our final sample, 37% of the individuals in the ALC cohort meeting the DSM-IV criteria for lifetime alcohol abuse, also met the DSM-IV criteria for lifetime alcohol dependence. In the CIDI interview, respondents also noted age of ALC onset and the amount of drinks per day (1 drink = one shot glass (hard liquor), or one mixed drink (hard liquor), or one glass of wine, or one 12 oz. of beer/ale) when most drinking.

Analyses: A comparison of the demographic characteristics between ALC and CTRL cohorts was performed using *t*-tests and χ^2^ tests, as appropriate. A binary logistic regression analysis was used to evaluate the association between each of the depression dependent-variables (MDE, MDD, and PDD)—with ALC status as an independent variable (ALC vs. CTRL)—while controlling for differences in age, sex, and race as additional independent variables included in the model. This analysis was performed for each chronic pain cohort separately. In addition, separate binary logistic regression analyses were performed to further explore differences between men and women with similar dependent and independent variables. Bonferroni corrections for multiple comparisons were applied to reduce the chance of Type 1 error. To test for differences between ALC and CTRL groups in the age of onset of each depressive disorder, *t*-tests were performed to compare the age of onset of each depressive disorder between ALC and CTRL for each chronic pain condition separately.

Binary logistic regression analyses were performed to examine bi-directionality among chronic pain, ALC, and depressive disorders. In so doing, we combined all three depressive diagnoses into one factor (called Depression) to represent the participants with a history of any depressive disorder (MDE, MDD, or PDD). Similarly, another factor (called Chronic Pain) was created to represent participants with a history of both pain diagnoses (chronic back/neck and frequent/severe headaches). Then, a binary logistic regression model was used to examine how alcohol abuse as a dependent variable, was associated with chronic pain and depression as independent variables, while controlling for differences in age, sex, and race. Another binary logistic regression model was used to examine the association of chronic pain as the dependent variable, with ALC and depression as the independent variables, while again controlling for differences in age, sex, and race.

## 3. Results

Our sample included only individuals who had responded to CPES questions related to chronic pain, depressive disorders, and alcohol abuse, and met the CPES inclusion/exclusion criteria. Characteristics of the sample are presented in Table 1. Statistical comparisons were made between ALC and CTRL groups. Demographic information for the total sample and the chronic pain group is included for descriptive purposes. 

As Table 1 indicates, the majority of individuals in both the ALC and CTRL cohorts reported no history of chronic pain (ALC, 69.1%; CTRL, 74.3%). However, chronic back/neck problems were reported at a higher rate (ALC, 18.3% vs. CTRL, 11.5%) and at an earlier onset (ALC, 25.1 years vs. CTRL, 28.1 years) in the ALC cohort. In comparison, absence of a history of depressive disorders was less common in the ALC group compared to the CTRL group (ALC, 31.3% vs. CTRL, 65.1%). The individuals in the ALC cohort were slightly younger, and had more men, and fewer Asians than the CTRL cohort. Household income levels were comparable between the two cohorts. While the overall distribution of education levels was similar between the two cohorts, there were fewer individuals in the ALC cohort who had 16 years or more education. 

Table 2 provides results of the regression analyses for each depressive disorder, comparing differences between the ALC and CTRL cohorts with and without chronic pain conditions.

**Figure 2:**
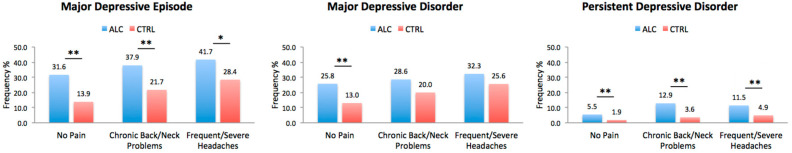
Depressive Diagnoses in ALC vs. CTRL in the Presence or Absence of Chronic Pain. The plots represent the lifetime incidence of depressive disorders in individuals with and without chronic pain (No Pain, Chronic Back/Neck Problems, or Frequent/Severe Headaches) with or without a history of alcohol abuse. CTRL refers to individuals with no history of alcohol abuse. Abbreviations: ALC, Alcohol Abuse Cohort; CTRL, Control Cohort. * *p* < 0.0166, ** *p* < 0.0033 adjusted thresholds with Bonferroni correction for three comparisons for the three depressive disorders.

The incidence of depressive disorders was significantly higher in ALC cohort even in the absence of any chronic pain disorders. In the presence of chronic back/neck problems, ALC individuals still had significantly higher incidence of MDD, MDE, and PDD compared to CTRL individuals. Differences between the ALC and CTRL cohorts remained significant for MDE and PDD with respect to severe/frequent headaches, but not for MDD. The comparisons are presented in Figure 2.

**Figure 3:**
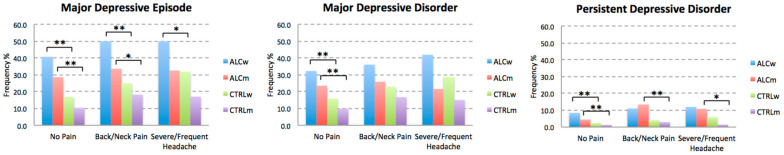
Breakdown of Depressive Disorders by Sex. The plots represent the lifetime incidence of depressive disorders in individuals with no chronic pain (i.e., No Pain, Chronic Back/Neck Problems, or Frequent/Severe Headaches) with or without a history of alcohol abuse. CTRL refers to individuals with no history of alcohol abuse. Abbreviations: ALCw, women with alcohol abuse history; ALCm, men with alcohol abuse history; CTRLw, control women; CTRLm, control men. * *p* < 0.0166, ** *p* < 0.0033, adjusted thresholds with Bonferroni correction for three comparisons for the three depressive disorders.

We also explored the breakdown of incidence by sex, and the results are presented in Figure 3. Overall, we found that the incidence of depressive disorders was the highest among ALC women and the lowest among CTRL men. PDD affected the fewest number of individuals in our sample. However, PDD was higher in ALC women than in ALC men, in both groups with no history of chronic pain. The incidence of PDD was comparable in ALC men and ALC women with a history of back/neck pain or severe headaches.

**Figure 4:**
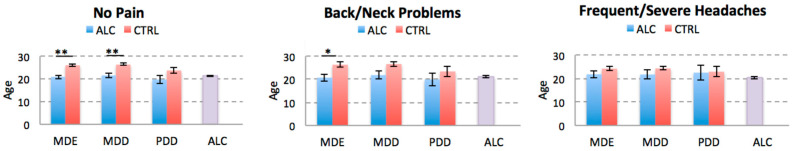
Comparison of the Age of Onset of Depressive Disorders. The plots represent the average age of onset of each depressive disorder for the ALC and CTRL cohorts stratified for the history of chronic pain. For comparison, average age of alcohol abuse onset is also presented (last column). Abbreviations: ALC, alcohol abuse; MDE, Major Depressive Episode; MDD, Major Depressive Disorder; PDD, Persistent Depressive Disorder. * *p* < 0.0166, ** *p* < 0.0033 adjusted thresholds with Bonferroni correction for three comparisons for the three pain disorders.

We also compared ALC and CTRL cohorts on age of onset of depressive disorders for each pain condition. We found that independently of the presence or absence of chronic back/neck pain, the age of onset of MDE was significantly younger in the ALC individuals, but it was comparable to the age of ALC onset. The age of onset of MDD was younger in the ALC cohort in the No Pain group compared to the CTRL cohort, but the difference in the back/neck problems cohort didn’t remain significant after correction for multiple comparisons. There were no significant differences between the age of ALC onset and any of the depressive diagnoses as confirmed by pair-wise t-test comparisons for each disorder. There were no significant differences in the age of onset of MDE, MDD, or PDD between the ALC and CTRL cohorts with frequent/severe headaches, which may in part be due to a sample size issue (see Discussion). The results of age of onset comparisons are shown in Figure 4.

The results of examining the bi-directional relationships between chronic pain with depression and ALC are presented in Table 3. While both chronic pain and depression are associated with increased odds of alcohol abuse, the odds are higher for depression (OR: 2.41 vs. 1.28). Depression is also associated with higher odds of chronic pain compared to alcohol abuse (OR: 1.69 vs. 1.28).

## 4. Discussion

Despite numerous reports on associations between chronic pain disorders, depressive disorders, and harmful drinking, it is not clear if the burden of a depressive disorder is similar in the presence or absence of ALC, in individuals who also have a chronic pain disorder. We had hypothesized that in the presence of chronic pain, the burden of depression would be similar for individuals with and without a history of ALC. In the present study, we found depressive disorders to be a high burden in ALC, independently of the presence or absence of chronic pain.

We also found a higher burden of MDE among ALC women compared to ALC men and CTRL men and women. Understanding the similarities and differences between the ALC and CTRL cohorts in depressive disorders is particularly intriguing because alcohol abuse is more prevalent in men than in women [31], while chronic pain disorders and depressive disorders tend to have a higher prevalence in women [32]. Moreover, in a longitudinal study, Boissoneault and colleagues [33], found that depression was predictive of alcohol problems only in women but not in men.

Additionally, we found that the onset of MDE in ALC group was younger than the CTRL group, whether or not they had chronic/severe back pain. Regarding the age of onset of the various conditions, we found that the onset of MDE in the ALC group was younger than in the CTRL group, whether or not either group experienced pain. We looked at the temporal relations between the ages of onset of each of the depressive disorders to determine if onset of ALC, preceded onset of MDE, MDD, or PDD. We found that there were no significantly different temporal patterns in onset of any of the depressive disorders relative to ALC onset. The comparability between ages of onset of alcohol abuse and depressive disorders may be suggestive of overlapping genetic predispositions for these disorders [34].

Finally, we showed bidirectional relationships between alcohol abuse, depression, and chronic pain. There is evidence for associations between changes in pain and depression levels for a number of different chronic pain conditions [35]. Treating depression may decrease pain conditions [36], especially if both pain and depression are most severe at the beginning of treatment [35]. Similarly, the synergistic effects of comorbid depression and ALC have been associated with greater severity for both disorders [37].

Dysfunction in the brain reward system seems to be considered as the prominent shared pathological link among these conditions [38]. However, it is not clear why despite the overlap between neural pathways underlying chronic pain and alcohol abuse, as well as the high comorbidity of both of those conditions with depression, the burden of depressive disorders is greater in people with ALC. Abnormalities in brain reward areas such as prefrontal cortex, anterior cingulate cortex, and insula, are consistently reported in association with chronic pain [18] and addictions [8,39]. Those same cortical regions are part of the depression neurocircuitry, but additionally, the ventral striatum, another reward system structure, has been reported to play a central role in depressive disorders [19]. The ventral striatum receives its primary input from those same cortical regions [40,41]. It is possible that differences between comorbid depression with ALC, and comorbid depression with chronic pain, may arise from differences in connectivity of cortical regions and the ventral striatum. Indeed, a recent meta-analysis by Ng and colleagues [42] has challenged the common notion that MDD is primarily linked to deficits within the reward circuitry, particularly the ventral striatum. Instead, they proposed that dysregulated corticostriatal connectivity may underlie reward-processing abnormalities in MDD. It is likely that the more prominent neuroplastic changes in these cortical regions due to direct or indirect effects of ALC may contribute to a stronger corticostriatal dysregulation and hence the higher likelihood of depression that we observed in association with ALC.

There are several limitations to consider in the present study. When selecting MDE, MDD, and PDD, we did not adjust for the presence of other comorbid psychiatric disorders. Furthermore, data on severity, duration, or interference with daily activities of the pain were only available for individuals who were suffering from pain up to 12-months prior to data collection. If the data were available, it would have been interesting to see if depression affected individuals with more severe or prolonged forms of chronic pain at a higher incidence, comparable to ALC individuals.

The sample size for our cohort with severe/frequent headaches was smaller than our other cohorts. This may account for the lack of statistical significance for the difference between the ALC and CTRL groups in age of onset of depressive disorders. To confirm this, we conducted additional analyses in which we included individuals who had a history of arthritis/rheumatism (one of our exclusionary criteria). By doing so, we increased the numbers of subjects in all three cohorts of no pain, chronic back/neck problems, and frequent/severe headaches. Accordingly, we found that the ages of onset of MDE, MDD and even PDD were consistently younger in the ALC cohort independently of the presence or absence of the chronic pain disorders compared to the control cohort.

Despite these limitations, our findings may have important implications for understanding the underlying factors contributing to depressive disorders in chronic pain patients. For example, it may be that a history of alcohol abuse (and perhaps additional forms of addictive behaviors) may play a pivotal role in explaining depressive disorders in at least a subset of individuals suffering from chronic pain disorders. Moreover, the presence of chronic pain disorders may delay seeking treatment for ALC, because depression or any other mood disorders stemming from alcohol abuse could be concealed by the presence of chronic pain [43]. Since physical pain is thought to be a potential predictor for relapse in individuals who are in recovery, it may be essential to approach treatment of pain as a strategy to also reduce the risk of relapse [44]. Remission of alcohol abuse could also complicate the treatment of chronic pain disorders, as it might increase the chances of depression remission in ALC patients with comorbid depression [45].

## 5. Conclusions

The findings of this study indicated that the incidence of MDE carries a substantial burden in ALC men and women, independently of whether or not they suffer from chronic back/neck pain or frequent/severe headaches. We also found that, although the incidence of PDD was comparable in men and women with ALC, and significantly higher than in CTRL men and women, the incidence of MDE was higher in ALC women independently of the presence or absence of chronic pain. The age of onset of depressive disorders, independently of pain status, was younger for ALC individuals. We also found bidirectional associations between chronic pain, depression, and ALC. Results of this study may play a pivotal role in explaining the incidence of depressive disorders in individuals suffering from chronic pain disorders and who have an ALC history. Routine screening for current or past drinking history [46] by primary care physicians is recommended. Such screening can assist in understanding the relationships between ALC and depression as comorbidities in association with chronic pain disorders, thereby tailoring treatment strategies to enhance positive outcomes [47].

## Figures and Tables

**Table 1:** Characteristics of the Sample.

	Missing	Total Sample (*n* = 6765)	Chronic Pain (*n* = 1777)	ALC (*n* = 764)	CTRL (*n* = 6001)	*p*-Value (ALC v. CTRL)
Age (mean ± *SD*)	0	38.3 ± 14.3	38.0 ± 13.5	36.7 ± 12.3	38.5 ± 14.5	<0.001 *
Sex (*n*, %)	0					<0.001 *
Male		3227 (47.7%)	683 (38.4%)	548 (71.7%)	2679 (44.6%)	
Female		3538 (52.3%)	1094 (61.6%)	216 (28.3%)	3322 (55.4%)	
Race (*n*, %)	0					<0.001 *
Caucasian		2395 (35.4%)	786 (44.2%)	427 (55.9%)	1968 (32.8%)	
Black		438 (6.5%)	127 (7.1%)	51 (6.7%)	387 (6.4%)	
Hispanic		2199 (32.5%)	537 (30.2%)	208 (27.2%)	1991 (33.2%)	
Asian		1649 (24.4%)	301 (16.9%)	56 (7.3%)	1593 (26.5%)	
Other		84 (1.2%)	26 (1.5%)	22 (2.9%)	62 (1%)	
Household Income (*n*, %)	15					0.085
≤$19,999		1540 (22.8%)	384 (5.7%)	171 (22.4%)	1369 (22.8%)	
$20,000–$49,999		1954 (28.9%)	509 (7.5%)	235 (30.8%)	1719 (28.6%)	
$50,000–$99,999		1944 (28.8%)	560 (8.3%)	231 (30.2%)	1713 (28.5%)	
≥$100,000		1312 (19.4%)	321 (4.8%)	123 (16.1%)	1189 (19.8%)	
Education (*n*, %)	0					<0.001 *
0–11 years		1280 (18.9%)	300 (4.4%)	154 (20.2%)	1126 (18.8%)	
12 years		1737 (25.7%)	465 (6.9%)	232 (30.4%)	1505 (25.1%)	
13–15 years		1853 (27.4%)	524 (7.7%)	224 (29.3%)	1629 (27.1%)	
≥16 years		1895 (28%)	488 (7.2%)	154 (20.2%)	1741 (29%)	
Pain History (*n*, %)	0					<0.001 *
No Chronic Pain		4988 (73.7%)	n/a	528 (69.1%)	4460 (74.3%)	
Chronic Back/Neck Problems		830 (12.3%)	830 (47%)	140 (18.3%)	690 (11.5%)	
Frequent/Severe Headaches		947 (14%)	937 (53%)	96 (12.6%)	851 (14.2%)	
Pain Onset (mean ± *SD*)						
No Chronic Pain	n/a	n/a	n/a	n/a	n/a	
Chronic Back/Neck Problems	8	27.6 ± 13.2	27.6 ± 13.2	25.1 ± 10.6	28.1 ± 13.6	<0.01 *
Frequent/Severe Headaches	35	21.1 ± 10.7	21.1 ± 10.7	20 ± 9.8	21.2 ± 10.8	0.304
Depression (*n*, %)	0					<0.001 *
MDE		1270 (18.8%)	485 (21.1%)	260 (34%)	1010 (16.8%)	
MDD		1144 (16.9%)	427 (18.6%)	207 (27.1%)	937 (15.6%)	
PDD		208 (3.1%)	96 (4.2%)	58 (7.6%)	150 (2.5%)	
None		4143 (61.2%)	1286 (56.1%)	239 (31.3%)	3904 (65.1%)	
Alcohol Abuse (mean ± *SD*)						n/a
Onset	0	21.2 ± 7	20.8 ± 6.3	21.2 ± 7	n/a	n/a
Abuse Duration	5	8.5 ± 8.3	8.7 ± 8	8.5 ± 8.3	n/a	n/a
Years Since Last Abuse	5	6.9 ± 8	7.3 ± 7.9	6.9 ± 8	n/a	n/a
Drinks/Day in Years w/Most Drinking	131	8.7 ± 6.7	5.4 ± 4.5	8.7 ± 6.7	n/a	n/a

* Statistically significant; *n*: number of individuals; SD: standard deviation; n/a: not applicable.

**Table 2:** Odds Ratios for the Association Between Depression and Alcohol Abuse in the Presence or Absence of Chronic Pain.

Factor	Odds Ratio	Confidence Interval for the Odds Ratio		Significance
		Lower	Upper	
Major Depressive Episode				
No Pain	2.54	2.04	3.16	<0.001 **
Chronic Back/Neck Pain	2.11	1.40	3.19	<0.001 **
Frequent/Severe Headaches	1.99	1.25	3.16	0.004 *
Major Depressive Disorder				
No Pain	2.04	1.63	2.57	<0.001 **
Chronic Back/Neck Pain	1.56	1.01	2.42	0.045
Frequent/Severe Headaches	1.51	0.93	2.45	0.092
Persistent Depressive Disorder				
No Pain	2.82	1.77	4.48	<0.001 **
Chronic Back/Neck Pain	3.10	1.61	5.94	0.001 **
Frequent/Severe Headaches	3.24	1.54	6.82	0.002 **

* *p* < 0.0166, adjusted threshold of 0.05 with Bonferroni correction for three comparisons. ** *p* < 0.0033, adjusted threshold of 0.01 with Bonferroni correction for three comparisons.

**Table 3:** (a) Odds ratios for the association of alcohol abuse with chronic pain and depression; (b) Odds ratios for the association of chronic pain with alcohol abuse and depression.

Factor	Odds Ratio	Confidence Interval for the Odds Ratio		*p*-Value
		Lower Limit	Upper Limit	
(a)				
Depression	2.41	2.02	2.87	<0.001
Chronic Pain	1.28	1.07	1.52	0.006
(b)				
Alcohol Abuse	1.28	1.08	1.53	0.005
Depression	1.69	1.48	1.93	<0.001

The three depressive diagnoses were combined into one factor (Depression) to represent the participants with a history of any depressive disorder (MDE, MDD, or PDD). Chronic Pain refers to the participants with a history of both pain diagnoses (chronic back/neck and frequent/severe headaches).
